# Bayesian Modeling of the Impact of HBOT on the Reduction in Cytokine Storms

**DOI:** 10.3390/jcm14041180

**Published:** 2025-02-11

**Authors:** Natalia Jermakow, Klaudia Brodaczewska, Jacek Kot, Arkadiusz Lubas, Krzysztof Kłos, Jacek Siewiera

**Affiliations:** 1 Department of Hyperbaric Medicine, Military Institute of Medicine, National Science Institute, Szaserów 128, 04-141 Warsaw, Poland; jacek.siewiera@gmail.com; 2 The Laboratory of Molecular Oncology and Innovative Therapies, Military Institute of Medicine, National Science Institute, Szaserów 128, 04-141 Warsaw, Poland; kbrodaczewska@wim.mil.pl; 3 National Centre for Hyperbaric Medicine, Institute of Maritime and Tropical Medicine, Medical University of Gdansk, Powstania Styczniowego 9B, 81-519 Gdynia, Poland; jkot@gumed.edu.pl; 4 Department of Internal Diseases Nephrology and Dialysis, Military Institute of Medicine, National Science Institute, Szaserów 128, 04-141 Warsaw, Poland; alubas@wim.mil.pl; 5 Department of Infectious Diseases and Allergology, Military Institute of Medicine, National Science Institute, Szaserów 128, 04-141 Warsaw, Poland; kklos@wim.mil.pl

**Keywords:** HBOT, COVID-19, immune response

## Abstract

Since the initial identification of SARS-CoV-2 infections, numerous clinical challenges have arisen, revealing both acute and long-term effects associated with COVID-19. These effects impact various systems within the body, including the respiratory, cardiovascular, and nervous systems. **Background/Objectives**: This study aimed to investigate the immunological and inflammatory parameters in patients with severe COVID-19 and evaluate the effects of hyperbaric oxygen therapy (HBOT) on these parameters. **Methods**: This study enrolled thirty patients from the Military Medical Institute—National Research Institute in Warsaw, who were hospitalized for SARS-CoV-2 infection. Patients were screened for eligibility based on pre-defined inclusion criteria. The subjects were randomly assigned to one of two groups: hyperbaric oxygen therapy (HBOT) or a control group. Immune profiling was performed, measuring cytokine concentrations and leukocyte subpopulations in serum samples. Outcomes were assessed using Bayesian modeling. **Results**: Bayesian regression analysis confirmed previous findings, indicating that HBOT may reduce inflammatory cytokine levels while improving oxygen saturation (SpO_2_) in patients with moderate and severe COVID-19. Moreover, the analysis suggested a higher probability of HBOT success in modulating the immune response and reducing inflammatory parameters, particularly in T lymphocyte subpopulations. **Conclusions**: Hyperbaric oxygen therapy (HBOT) may serve as an effective adjunctive treatment for patients with COVID-19 by enhancing oxygen saturation and modulating the immune response. Further studies are needed to elucidate the underlying mechanisms of HBOT on inflammatory and immunological parameters in COVID-19 patients.

## 1. Introduction

Since May 2020, there have been more than 770 million confirmed cases of SARS-CoV-2 infection worldwide, resulting in approximately 7 million deaths. Although it is no longer a significant global problem, the infection has still been detected in 130,000 people, and almost 3000 deaths have been recorded as of the turn of 2024/2025 [1]. Despite the current lack of significant threat posed by the novel coronavirus disease (COVID-19), the emergence of new cases and fatalities due to infection with the recently identified JN.1+JN.1.* variant responsible for the most significant number of infections at the turn of 2024/2025 [2] underscores the necessity for continued vigilance.

Since the initial identification of cases of SARS-CoV-2 infection, numerous clinical challenges have been encountered, both in the context of ongoing infection and in the subsequent acute and long-term effects [3,4]. These effects encompass a range of systems, including the respiratory, cardiovascular, and nervous systems, as well as cognitive processes [5,6,7].

The SARS-CoV-2 virus enters lung alveolar cells via the angiotensin-converting enzyme 2 (ACE2) receptor, thereby negatively regulating vasoconstriction, cell proliferation, and inflammation [8]. Consequently, the most prominent respiratory symptoms are observed in patients with developed symptoms. These primarily include cough, shortness of breath, and pneumonia with acute respiratory distress syndrome (ARDS). Therefore, the first-line treatment for patients with respiratory failure due to SARS-CoV-2 is administering oxygen via a high-flow nasal cannula [9].

At the immunological level, studies have described excessive activation of immune cells and blood clotting in patients, accompanied by tissue inflammation [10,11]. Several studies have demonstrated that patients with moderate to severe cases of coronavirus disease 2019 (COVID-19) exhibited an impaired T-cell response and an imbalance of major T helper lymphocyte (Th) subpopulations [12,13,14,15]. In severe cases, this could result in an imbalance of the immune system. The excessive and uncontrolled production of pro- and anti-inflammatory cytokines has been identified as a key factor contributing to both a worse prognosis of SARS-CoV-2 infection and acute respiratory distress syndrome [16,17]. This includes a significant role in the excessive production of early pro-inflammatory cytokines, including TNF-α, IL-6, IL-1β, and IFN-γ [18]. The precise relationship between cytokine levels and the course of infection remains unclear.

Nevertheless, in the acute phase of infection, the key roles are played by interferon-gamma (IFN-γ), interleukin 1 alpha (IL-1α), interleukin 7 (IL-7), interleukin 9 (IL-9), interleukin 10 (IL-10), granulocyte colony-stimulating factor (G-CSF), fibroblast growth factor (FGF), granulocyte-macrophage colony-stimulating factor (GM-CSF), tumor necrosis factor-alpha (TNF-α), and vascular endothelial growth factor (VEGF). Additionally, chemokines CXCL-8 (IL-8), CXCL-10 (IP-10), CCL-2 (MCP-1), CCL-3 (M1α), and CCL-4 (MIP-1β) were identified in a previous study [19]. The elevated levels of IL-1β, IFN-γ, MCP-1, and IP-10 observed in patients with severe SARS-CoV-2 infection may stimulate the activation of Th1 lymphocytes, thereby triggering a cascade of cytokines and exacerbating the inflammatory response [20]. In a separate study, elevated levels of pro-inflammatory cytokines, including IL-6, IL-8, IL-2R, TNF-α, and anti-inflammatory IL-10, were observed in the serum of patients with severe disease progression [21].

The precise mechanism by which a cytokine storm is triggered in severe cases of coronavirus disease 2019 (COVID-19) remains unclear. The data indicate that a reduction in the number of lymphocytes, particularly Tc cells, which function by eliminating infected cells [22], in conjunction with neutrophils that mediate the cytokine storm [23], may be a pivotal factor in the pathogenesis of SARS-CoV-2 infection. Recent reports of pathological findings in autopsied patients with confirmed diagnoses of SARS-CoV-2 infection have noted the presence of neutrophil infiltration in affected tissues [24,25]. Similarly, it has been reported that increases in neutrophils and reactive oxygen species are observed in patients with severe SARS-CoV-2 infection [26]. The production of excessive reactive oxygen species (ROS) by neutrophils can propagate local inflammation, ultimately leading to systemic dissemination [27]. The clinical efficacy of hyperbaric oxygen (HBO) therapy has been demonstrated in the reversal of local hypoxia and subsequent reduction in inflammatory processes [28,29,30]. Additionally, an increase in reactive oxygen species (ROS) production has been observed during HBOT-induced hyperoxia [31]. Nevertheless, the findings indicate that participation in multiple HBOT sessions diminishes the capacity of neutrophils to generate ROS while not elevating plasma cytokine concentrations [32,33]. Furthermore, the temporary increase in ROS levels resulting from HBOT has led to its successful application in treating acute and chronic wounds, diabetic foot ulcers, and infectious diseases [34,35,36,37,38]. In recent years, there has been a notable increase in the number of clinical studies that have provided evidence supporting the efficacy of hyperbaric oxygen therapy (HBOT) in modifying the immune system, particularly in the context of soft tissue infections (NSTIs) and non-specific intestinal inflammations. Furthermore, there is evidence that hyperbaric oxygen therapy is effective in the treatment of nonspecific intestinal inflammations [39] (pp. 55–65) and in bowel disease [40]. Some studies have proposed hyperbaric oxygen therapy (HBOT) as an effective method of reversing acute complications and treating patients with long-term COVID-19 symptoms [5,6]. From the outset of the SARS-CoV-2 pandemic, there has been a focus on the efficacy of hyperbaric oxygen therapy (HBOT) in improving oxygen saturation in patients with coronavirus disease 2019 [41]. Nevertheless, the application of HBOT for transient improvement in patient oxygenation is not advised [42].

In addition, there are an increasing number of scientific reports proving the efficacy of HBOT in improving saturation in COVID-19 patients, with a clinical trial protocol published in 2022 to evaluate the safety and efficacy of HBOT in patients with COVID-19 [43]. The study proved an increase in SpO_2_ in COVID-19 patients with severe hypoxemia without significant adverse effects. These results are consistent with a study we published previously [44]; despite the objective of our report being a reduction in mortality, the statistical decrease in mortality was not significant. A growing number of studies are demonstrating the benefits of HBOT, in patients with both active infection and long-term COVID-19 [17,45,46,47]. Considering the mounting evidence attesting to the efficacy of hyperbaric oxygen therapy (HBOT) in treating patients with the novel coronavirus disease (2019-nCoV), this study broadens the analysis of immunological and inflammatory parameters. It also implements Bayesian modeling to evaluate different scenarios and compounds on which HBOT exerts a significant influence, with a high probability of success. The objective was to evaluate the probability of reducing inflammatory cytokine parameters, particularly the proportion of T lymphocyte subpopulations and the concentrations of pro- and anti-inflammatory cytokines.

## 2. Materials and Methods

The full description of the following sections can be found in our previous publication [44].

### 2.1. Patient Characteristics

Thirty patients of the Military Medical Institute—National Research Institute in Warsaw, aged 24 to 78 (6 women; mean age 55 ± 13.4 years) and hospitalized for SARS-CoV-2 infection between 1 March 2021 and 3 February 2022, participated in the study [44].

All patients who met the inclusion and did not meet the exclusion criteria were randomly assigned to HBOT or control groups. Two patients were excluded because they did not meet the inclusion criteria. Fourteen patients were assigned to the HBOT group, and fourteen patients were placed in the control group. There were three deaths in the control group, and no adverse events (AEs) leading to the discontinuation of any single HBOT session were observed in the HBOT group. All patients received subcutaneous anticoagulants and corticosteroids as part of their treatment. Twenty-seven patients (including two excluded from the study) were given antibiotics, five received remdesivir (HBOT: *n* = 3; control: *n* = 2), and one patient in the HBOT group was treated with tocilizumab.

### 2.2. Study Design

Patients were screened daily by a researcher trained in anesthesiology, intensive care, and hyperbaric medicine. Those who met the inclusion criteria and had no exclusion criteria were informed about the study’s purpose. After providing written informed consent, participants were randomized into either the HBOT or control group. No patient underwent HBOT or had biological material collected prior to signing the consent form.

In contrast to the control group, the HBOT group underwent five hyperbaric sessions. HBOT sessions were performed at 2.5 ATA for 75 min, including 5 min of compression, 60 min of 100% oxygen breathing via individual oxygen helmets, and 10 min of decompression, adjusted for medical personnel. Sessions were conducted daily for up to 5 sessions.

Before and following each session, an arterial blood gas test was conducted, and vital signs were documented. Additionally, blood samples were obtained for comprehensive biochemical and hematological analyses. Immunological tests were performed on the first, fifth, and tenth days.

### 2.3. Detection of Cytokines and Growth Factors in Serum

IL-12p70, TNF-α, IL-4, IL-10, IL-1β, Arginase, TARC, IL-1RA, IL-12p40, IL-23, IFN-γ, and IP-10 in serum were measured by the LEGENDPlex bead method (HU Macrophages/Microglia Panel (13-plex), Biolegend, USA). Serum samples were thawed on ice, centrifuged at 1000× *g* for 10 min at 4 °C, diluted according to the manufacturer’s protocol, and incubated with capture beads for 2 h at RT with shaking. They were then washed twice and incubated with detection antibodies for 1 h at room temperature with shaking. Detection was performed by incubating SA-PE for 30 min at room temperature with shaking, and after washing, beads were acquired on a CytoFLEX Flow Cytometer (Beckman Coulter, USA) and analyzed by CytExpert v.2.3.0.84 software (Beckman Coulter, USA) according to the manufacturer’s instructions. The concentration of factors was calculated relative to the standard curve using the recombinant proteins provided in the kit.

### 2.4. T-Cell Immunophenotyping

**Figure 1:**
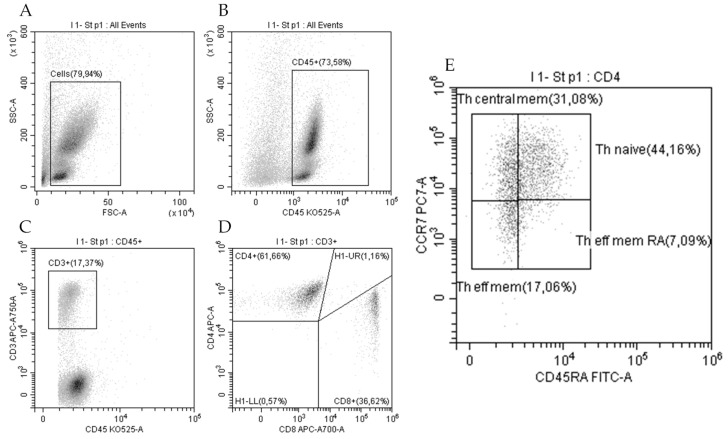
Gating strategy for lymphocytes subsets: all cells (**A**), CD45+ leucocytes (**B**), T lymphocytes CD45+CD3+ (**C**), Th CD45+CD3+ CD4+ (CD4+), and Tc CD45+CD3+ CD8+ (CD8+) lymphocytes (**D**). Then, Th cells were identified as Th effector memory re-expressing CD45RA (Th eff mem RA; CD62L–CD45RA+), Th-naïve (CD62L+CD45RA+), Th effector memory (CD62L−CD45RA; Th eff mem), and Th central memory (Th central mem; CD62L+CD45RA–) (**E**).

Peripheral blood samples were collected in an EDTA-anticoagulated tube (Blood Collection tube Vacutainer^®^, BD, Warsaw, Poland). Whole blood was used for immunofluorescence staining of extracellular markers, and 100 µL of blood was stained with anti-CD4-APC (# IM2468, Beckman Coulter), anti-CD8-APC-AF700 (# B49181, Beckman Coulter), anti- CD3-APC-AF750 (# A94680, Beckman Coulter), and anti-CD45-KrO (# B36294, Beckman Coulter) for 30 min at RT. As a negative control, we used unstained blood; single staining was used for compensation settings and gating strategy. Erythrocytes were lysed for 15 min at RT using BD FACS TM Lysing Solution (BD Bioscience, USA) and washed twice in PBS. Then, cells were fixed with Cytofix fixation buffer for 5 min (BD Bioscience, USA). Cells were acquired on a CytoFLEX Flow Cytometer (Beckman Coulter, USA) and analyzed by CytExpert v.2.3.0.84 software (Beckman Coulter, USA). Debris was gated out on an FCS/SSC dot plot, and 30,000 events in the “Cells” gate were analyzed (Figure 1).

### 2.5. Model Construction and Validation

The analysis was conducted in RStudio (version 2023.06.1; R version 4.3.1), with the brms package (version 2.20.1) employed for this purpose. Five Bayesian multiple regression models were constructed. Any missing values in the output variable were handled with the last observation carried forward (LOCF) method. The first model considers only the interaction between the day and group factor and the random factor (patient ID). The second model includes the day factor as the grouping factor of the group factor and the random factor (patient ID). The third model examines the interaction of the day x group factor and the random factor of the day as the grouping factor. Patient ID was included as a random effect in the model. 4. A model with the day factor as the grouping factor of the “group” factor and the random factor of the patient ID was used. 5. A logistic model with the calculated change between days 10/5 and 5/1 within the group and a random factor of the patient ID was used.

Given the considerable variability observed, the raw values in models 1–4 were transformed into Z-values and assigned a Gaussian family. In Model 5, the family parameter was set to Bernoulli. In each model, a normal distribution with mean M = 0 and standard deviation SD = 1 was used as the prior, and the models were run with four Markov chains of 4000 iterations. This is the first study to use HBOT in COVID-19 patients; it is a weak prior criterion based on data distribution only.

The model with the optimal fit to the data was selected using the leave-one-out cross-validation (LOO) method with the LOO criterion, as well as the expected log pointwise predictive density (ELPD) difference, which was used to assess the overall predictive distribution of each of the first four models. Model 5, however, could not be included in this comparison due to its distinct design. Additionally, the LOO weights of each model were evaluated. Cross = validation selected model 3 in the most cases as the best fit for the data (the lowest ELPD difference values or equal to zero).

## 3. Results

### 3.1. Patients

No statistically significant differences were found in the prevalence of comorbidities between the HBOT and control groups (Chi^2^ = 0; *p* > 0.999). There were no significant differences between the HBOT and control groups regarding age (56.07 ± 14.02 vs. 52.08 ± 13.51; U = 83.5; *p* = 0.52) or sex distribution (Chi^2^ = 0; *p* > 0.99). Baseline clinical responses, as measured by the National Early Warning Score (NEWS), were comparable in both groups (HBOT: M = 2.35, SD = 1.3; control: M = 2.5, SD = 1.1; *p* = 0.964), with similar results recorded at the final HBOT measurement (HBOT: M = 1.72, SD = 0.99; Control: M = 2.36, SD = 1.8; *p* = 0.962).

### 3.2. Bayesian Model Results

**Figure 2:**
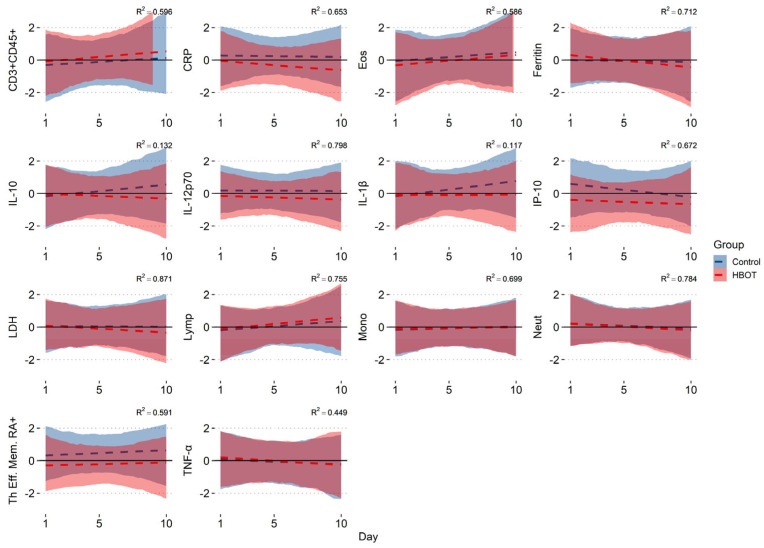
Bayesian regression model; the figure shows conditional effects with the mean value of the regression coefficient (dashed line) along with 95% confidence intervals and Bayesian R^2^ measures indicating the goodness of fit of the model, respectively: CD3+ (% CD45+) lymphocyte population; C-Reactive Protein (CRP); Eosinophiles (Eos); Ferritin, Interleukin 10 (IL-10); Interleukin 12 (IL-12 p70); Interleukin 1 beta (IL-1β); Interferon gamma-induced protein (IP-10/CXCL10); Lactate dehydrogenase (LDH); Lymphocytes (Lymph); Monocytes (Mono); Neutrophils (Neut); RA+ Th effector memory lymphocytes (Th Eff. Mem. RA+); tumor necrosis factor alpha (TNF-α).

In the input measure, control and HBOT subjects did not differ significantly in the concentrations of the analyzed cytokines and leukocyte subpopulations (Mann–Whitney analysis: *p* > 0.05), except for IP-10 (*p* = 0.0018). Bayesian regression analysis showed consistent values with our previous publication, i.e., a decrease in the HBOT group in CRP −0.077 (95% CI: −0.122; −0.027), Ferritin −0.066 (95% CI: −0.107; −0.024), and LDH −0.047 (95% CI: −0.083; −0.014); an increase in both groups in Eos (HBOT: 0.073 [95% CI: 0.019; 0.125]; C: 0.068 [95% CI: 0.015; 0.121]) and Lymp (HBOT: 0.074 [95% CI: 0.026; 0.122]; C: 0.059 [95% CI: 0.012; 0.109]); a decrease in Neutr (HBOT: −0.046 [95% CI: −0.079; −0.015]; C: −0.034 [95% CI: −0.066; −0.003]); and an increase in the CD3+(% CD45+) lymphocyte population in the HBOT group of 0.071 (95% CI: 0.023; 0.122). In addition, the HBOT group showed a probability of a decrease over time in cytokine levels of TNF-α: 0.048 (95% CI: −0.098; −0.0002), IL-10: −0.085 (95% CI: −0.154; −0.017), and IP-10/CXCL10: −0.080 (95% CI: −0.129; −0.031); a decrease in IL-12p70: −0.026 (95% CI: −0.051;−0.0007); and in the control group, an increase in the percentage of RA+ Th effector cells: 0.047 (95% CI: 0.0006; 0.095), IL-1β: 0.094 (95% CI: 0.034; 0.158), and Mono 0.021 (95% CI: 0.003; 0.039) (Figure 2). In the remaining variables, the probabilities were not significant (95% CI exceeded 0), so there was no basis to conclude any probability of change over time in either group in IL-6, IFN-γ, IL-12p40, IL-1R, IL-23, IL-4, TARC, CD8+, Tc subpopulations, Th central memory, Th effector memory, Th naïve, or WBC.

## 4. Discussion

The present study demonstrates that hyperbaric therapy effectively influences immune dysregulation in patients with SARS-CoV-2 infection. The results indicated a reduction in inflammatory mediators, including TNF-α, IL-10, IP-10, and IL12p70, associated with unfavorable patient outcomes when present at elevated levels. Furthermore, the absence of hyperbaric oxygen therapy (HBOT) was associated with an increased likelihood of an increase in interleukin-1 beta (IL-1β) levels within 10 days.

While there is a growing body of literature on the positive effects of hyperbaric oxygen therapy (HBOT) in patients infected with SARS-CoV-2, the majority of studies have focused on the positive effects on the respiratory system [17,40,42,46,48], including blood saturation, myocardial function [47], and cognitive function [48,49]. In the case of the short-term effects of HBOT, findings corroborate the safety and efficacy of hyperbaric oxygen therapy (HBOT) in treating COVID-19. Furthermore, these findings indicate that the time required to correct hypoxemia was shorter in patients who underwent HBOT [43]. A publication describing the effect of hyperbaric oxygen therapy (HBOT) on respiratory function and inflammatory parameters in patients requiring oxygen supplementation demonstrated that HBOT reduced the levels of lactate dehydrogenase (LDH), D-dimers, and endothelial adhesion molecules, including sVCAM-1, sICAM-1, sP-selectin, and inflammation-related proteins such as SAA and MPO. Among the cytokines tested, a significant difference was observed only in IL-15 [45].

Nevertheless, most extant studies concerning the utilization of hyperbaric oxygen therapy (HBOT) in SARS-CoV-2 infection concentrate on its deployment in managing the long-term consequences of coronavirus disease 2019 (COVID-19). In a study evaluating cognitive function in post-COVID-19 patients treated with HBOT [48], authors proved that administering hyperbaric oxygen therapy (HBOT) resulted in a statistically significant improvement in higher cognitive functions and attention. In a case series of ten patients following SARS-CoV-2 infection, Robbins et al. [49] proposed that HBOT may confer benefits concerning fatigue and cognitive function. They observed notable improvements in both cognitive function and physical and mental symptoms. In addition to this clinical study, cardiac function was also assessed. In another study involving post-COVID patients reporting cognitive problems affecting their quality of life, HBOT was demonstrated to reduce impaired systolic function (GLS) [47]. A growing body of clinical and scientific evidence suggests that hyperbaric oxygen therapy (HBOT) may be an effective method for improving the course of infection and reducing the risk of distant complications [3,4,46,47,48,49].

This study reveals that hyperbaric oxygen therapy (HBOT) has a significant effect on immune dysfunction in individuals afflicted with SARS-CoV-2. The findings showed a notable decrease in various inflammatory markers, including TNF-α, IL-10, IP-10, and IL-12p70, typically linked to poor patient outcomes when present at high levels. Furthermore, patients not receiving HBOT displayed a higher likelihood of increased interleukin-1 beta (IL-1β) levels within 10 days. Additionally, it has been shown that the probability of an increase in Th effector memory RA^+^ lymphocytes is higher in a control group. On the one hand, this may suggest that patients in this group have a more effective cell response, but the results suggest that, although HCs that have higher levels of RA+ memory effector, these cells do not predict a better outcome [50]. While a growing body of research supports the beneficial effects of HBOT, particularly concerning respiratory health, our results affirm its safety and effectiveness as a treatment option for COVID-19.

This study’s benefits consist of analyzing molecules involved in immune responses through sophisticated analytical techniques, and offering crucial insights into the interaction between HBOT and immune modulation, particularly when treatment commences early during the illness. This suggests that HBOT could be an effective supplementary therapy for enhancing patient recovery and easing complications associated with COVID-19. Future clinical trials are necessary to substantiate these findings and broaden the understanding of HBOT’s role in treating SARS-CoV-2 infections and their aftereffects. Limitations of this investigation include the relatively small participant group, which may limit the applicability of the results. Moreover, further studies are essential to confirm the long-term impact of HBOT on inflammatory indicators and immune reactions.

## 5. Conclusions

Our study’s results complement our previous findings and supplement them with new molecules, i.e., T lymphocyte subpopulations and pro-inflammatory molecules, using more complex probability-based statistical modeling. By analyzing TNF-α, IP-10/CXCL10, and IL-12p70, we show that the probability of a decrease in these factors, associated with a poor outcome, was observed in HBOT but not in the control group. These results suggest that hyperbaric oxygen therapy (HBOT) may improve the condition of patients with SARS-CoV-2 infection and reduce excessive immune cell activity, particularly when initiated early in the course of illness and probably only in the narrow spectrum of highly selected patients for whom other treatments have been ineffective or have been refused by patients. Bergersen et al. (2023) highlighted the increased severity of COVID-19 in high-risk patients with long-COVID symptoms persisting for up to 11 months despite the recovery of lung function [51]. Combined with our findings, which demonstrate disrupted immune homeostasis following COVID-19, HBOT appears to have the potential to restore immune balance and offer significant benefits for patients suffering from long-term COVID-19. While there is optimism regarding the potential role of HBOT in treating patients with SARS-CoV-2 infection, especially those with long-term complications from the virus, further clinical trials are necessary.

## Data Availability

The data presented in this study are available on request from the corresponding author upon reasonable request.
